# Fungitoxicity against *Botrytis cinerea* of a Flavonoid Isolated from *Pseudognaphalium robustum*

**DOI:** 10.3390/molecules16053885

**Published:** 2011-05-09

**Authors:** Milena Cotoras, Leonora Mendoza, Alexis Muñoz, Karen Yáñez, Paulo Castro, María Aguirre

**Affiliations:** Facultad de Química y Biología, Universidad de Santiago de Chile, Avenida Bernardo O’Higgins 3363, Santiago, Chile; Email: gotens666@gmail.com (A.M.); karennya@msn.com (K.Y.); paulo.castro@usach.cl (P.C.); maria.aguirre@usach.cl (M.A.)

**Keywords:** antifungal activity, *Botrytis cinerea*, 5,7-dihydroxy-3,8-dimethoxyflavone, *Pseudognaphalium robustum*, pro-oxidants

## Abstract

The fungotoxicity against *Botrytis cinerea* of a flavonoid isolated from *Pseudognaphalium robustum* was analyzed. Two absorption column chromatographies and one semipreparative thin layer chromatography were used to purify the active flavonoid. It was determined, by ^1^H-NMR spectroscopy and co-elution with standards in HPLC, that this compound was 5,7-dihydroxy-3,8-dimethoxyflavone (gnaphaliin A). To determine the fungitoxicity of the purified compound, the effect on *in vitro* mycelial growth and conidial germination was studied. The compound concentration that reduced mycelial growth by 50% was 45.5 μg/mL. This compound also partially affected conidial germination of *B. cinerea*, reduced oxygen consumption by germinating conidia and affected the integrity of plasma membrane. Finally, using cyclic voltammetry, it was shown that the purified flavone had a pro-oxidant effect.

## 1. Introduction

*Botrytis cinerea* (teleomorph *Botryotinia fuckeliana*) is a phytopathogenic fungus that infects more than 250 plant species worldwide. This fungus is difficult to control because it colonizes different plant organs such as flowers, fruits, leaves and stems, and it is most destructive on mature or senescent tissues producing serious damage during the storage of harvested fruits and vegetables [1]. 

Chemical control with synthetic fungicides is the main way to reduce diseases caused by *B. cinerea*, however the use of these fungicides has caused the development of fungicide-resistant *B. cinerea* strains and contamination of soil and water. The latter has generated a negative public perception toward the use of these fungicides in general [2]. Therefore, biodegradable chemicals such as secondary metabolites from plant are viewed as an alternative to synthetic fungicides.

Flavonoids form an interesting group of secondary metabolites due to their chemically diverse and biological properties [3]. Anti-inflammatory, anti-allergic, antimicrobial, anticarcinogenic, and antioxidant activities have been reported for these natural products [3]. Flavonoids can also act as pro-oxidants, depending on their concentration, free radical source, and the presence of transition metals [4]. 

The antifungal activity of these compounds has been reported [3,5]. Flavanones, flavans and lipophilic flavones have been associated with antifungal activity [6]. Sakuranetin, a flavonoid isolated from the surface of *Ribes nigrum,* inhibited germination of *B. cinerea* conidia [6]. On the other hand, three chalcones from the wood of *Bauhinia manca* affected mycelial growth of *B. cinerea* [6]. Resveratrol, a stilbene produced by grapes, inhibited the spread of *B. cinerea* infection [7]. 

In Chile, 14 species of plants of the genus *Pseudognaphalium* have been identified, among them *P. robustum*, which present a great variety of flavonoids in their resinous exudates [8,9]. Extracts obtained from resinous exudates of this plant partially reduced mycelial growth of *B. cinerea* [10]. Indeed, the flavone 5,7-dihydroxy-3,8-dimethoxyflavone has been reported as component of *P. robustum* resinous exudates [8,9]. This flavone, at 40 μg/mL, inhibited the growth of *B. cinerea* mycelia by 32% [10]. 

The aim of this work was to analyze if resinous exudate of *P. robustum* contains other flavonoids active against *B. cinerea*. For this, a bioassay-guided purification of flavonoids from these exudates was carried out. On the other hand, the effect of purified flavone on mycelial growth, conidial germination, oxygen consumption and membrane integrity of *B. cinerea* was evaluated. The pro-oxidant ability of the purified flavonoid was also determined.

## 2. Results and Discussion

**Figure 1:**
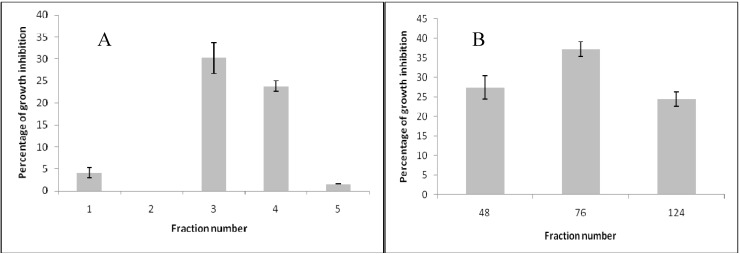
Effect on mycelial growth of *B. cinerea* of fractions obtained from column chromatography. Fractions (40 μg/mL) dissolved in dichloromethane from first (**A**) or second (**B**) column chromatography were added. Final solvent concentration was identical in the control and treatment assays. Each bar represents the mean of at least three independent experiments ± standard deviation.

Two absorption column chromatographies and one semipreparative thin layer chromatography were used to purify a flavonoid active against *B. cinerea* from the resinous exudates of *P. robustum.* Fraction 3 from the first column chromatography presented the highest antifungal activity against *B. cinerea* (Figure 1A). After 72 h of incubation, this fraction produced 30% inhibition of mycelial growth at 40 µg/mL. Fraction 3 was submitted to a second column chromatography and four fractions were obtained. Only, fractions 48, 76 and 124 showed effects on mycelial growth of *B. cinerea* (Figure 1B). 

**Figure 2:**
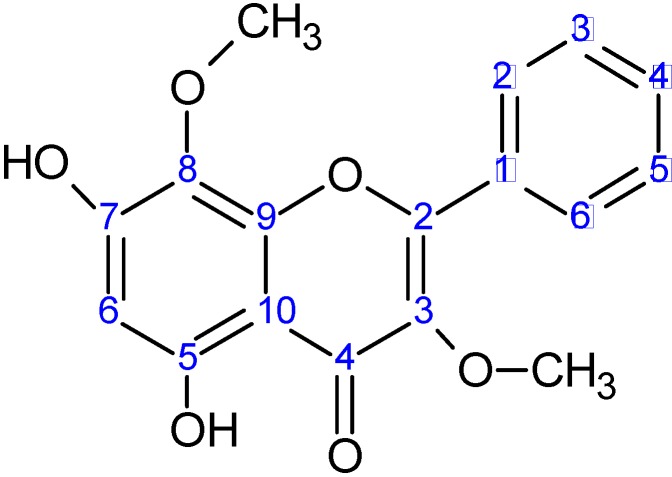
Structure of the compound 5,7-dihydroxy-3,8-dimethoxyflavone isolated from *P. robustum*.

Purification was continued with fraction 76, which was subjected to a semipreparative thin layer chromatography, yielding fraction F76-2 that corresponded to a pure compound that was identified as 5,7-dihydroxy-3,8-dimethoxyflavone (Figure 2) by comparison of its spectroscopic data (^1^H-NMR) with those reported in the literature [11,12] and co-elution with standards by HPLC. This compound, also called gnaphaliin A, has been isolated from other plants and it presents several biological activities [13,14]. 

The effect of the purified flavone on mycelial growth and conidial germination of *B. cinerea* was analyzed next. This compound inhibited mycelial growth, with an ED50 value of 45.5 µg/mL. This isolate is sensitive to iprodione, ED50 value for this commercial fungicide was 0.5 µg/mL.

**Figure 3:**
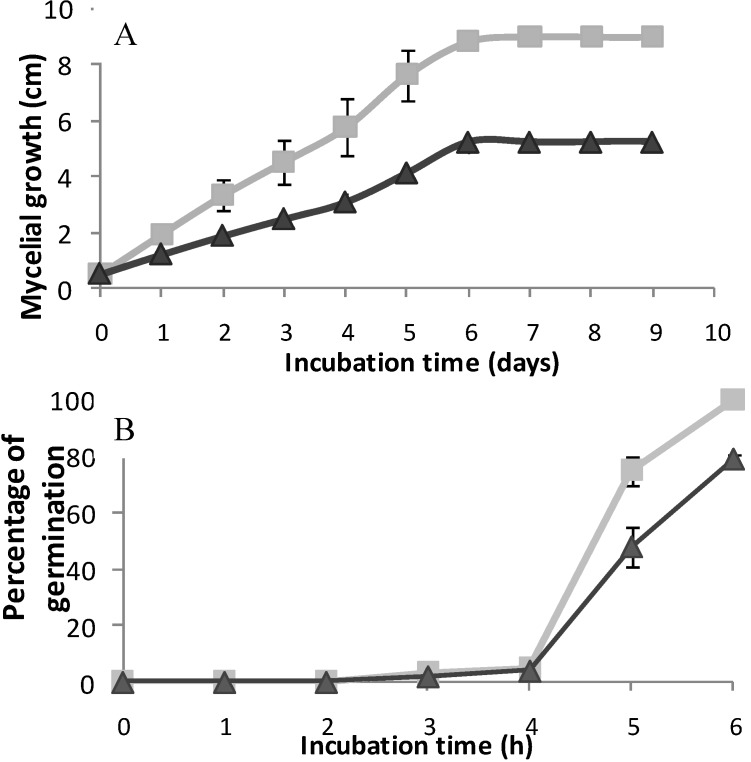
Effect of 5,7-dihydroxy-3,8-dimethoxyflavone on mycelial growth (**A**) and conidial germination (**B**) of *B. cinerea*. The compound was added dissolved in dichloromethaneat 40 μg/mL (▲). Final solvent concentration was identical in the control (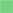) and treatment assays. Each point represents the mean of at least three independent experiments ± standard deviation.

In the presence of flavone at 40 µg/mL, mycelial growth stopped after six days of incubation without reaching maximum radial growth as control (Figure 3A). Biotransformation of this compound into a more toxic molecule might explain this observation. *B. cinerea* can modify compounds, mainly by hydroxylation, to more hydrophilic molecules [15]. In general, biotransformed products are less toxic. An exception is resveratrol, which is modified by a laccase from *B. cinerea* into a more toxic dimer [16]. 

On the other hand, flavone partially affected *B. cinerea* conidial germination (Figure 3B). At 40 µg/mL this compound inhibited germination by 30% after six hours of incubation. Some compounds that affect conidia germination act on mitochondrial respiration as strobilurin-type fungicides that block the electron transport chain and inhibit spore germination [17]. 

**Figure 4:**
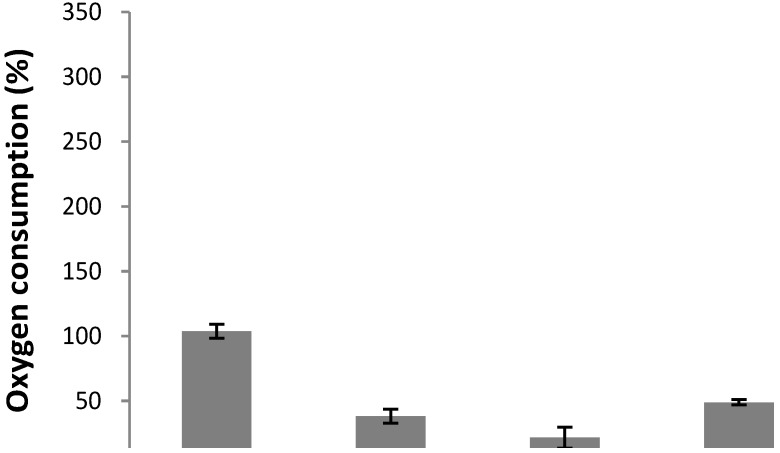
Effect of 5,7-dihydroxy-3,8-dimethoxyflavone at 40 or 80 μg/mL on oxygen consumption of germinating conidia from *B. cinerea*.. Flavone was added to a conidia suspension in dichloromethane. After 8 min of incubation, the percentages of inhibition in the presence of the compounds relative to the basal oxygen consumption were calculated. Final solvent concentration was identical in the control and treatment assays. CCCP (10 μg/mL) and KCN (650 μg/mL) were used as uncoupler or inhibitor control, respectively. Each bar represents the average of at least three independent experiments ± standard deviation.

Depending on their structural features, it has been reported that flavonoids can act as potent inhibitors of mitochondrial respiration or uncouplers [18]. Therefore, the effect of purified flavone on oxygen consumption by germinating conidia of *B. cinerea* was determined (Figure 4). At 40 µg/mL the compound inhibited 35.4% the oxygen consumption compared to the control. This effect was similar to that of KCN. 

**Figure 5:**
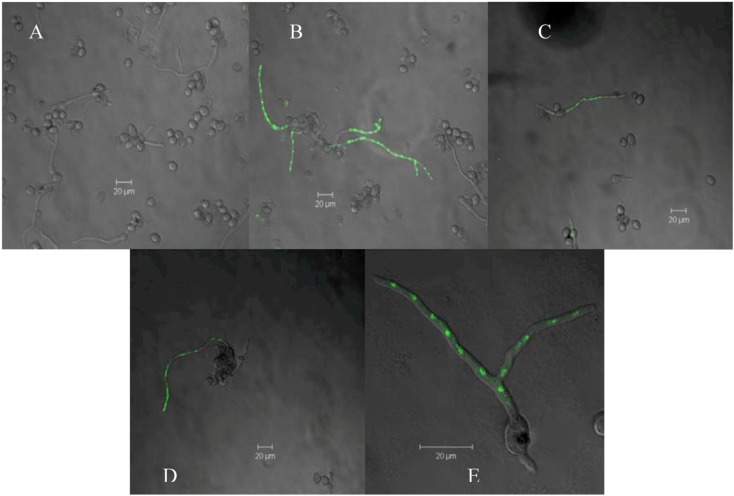
Effect of 5,7-dihydroxy-3,8-dimethoxyflavone on the membrane integrity of *B. cinerea.* Conidia, at a final concentration of 1 × 10^5^ conidia/mL, were incubated in liquid minimum medium in the presence of 8% (v/v) dichloromethane (**A**), 70% (v/v) ethanol (**B**), 80 μg/mL of 5,7-dihydroxy-3,8-dimethoxyflavone for 4 h (**C**) or 6 h (**E**) or 40 μg/mL of 5,7-dihydroxy-3,8-dimethoxyflavone for 6 h (**D**) hours. The fluorescence of *B. cinerea* hyphae stained with SYTOX Green was observed under a confocal microscope. These photographs are representatives of five independent experiments.

Regarding structural characteristics in antifungal flavonoids, it has been reported that one hydroxyl group and some degree of lipophilicity are important for antifungal activity [10,19,20]. Moreover, methoxyl substitution increased this activity [10,21]. On the other hand, characteristics such as the presence of a 2,3 double bond and a 4-carbonyl group, a low level of hydroxylation and *O*-methylation are associated with a significant increase of the toxicity of flavonoids on human leukemia cells [22]. These structural characteristics permit flavonoids to interact with cell membranes affecting mitochondrial functions [18]. The flavone identified in this study, 5,7-dihydroxy-3,8-dimethoxy-flavone, meets all these structural characteristics. By using SYTOX Green staining, it was demonstrated that 5,7-dihydroxy-3,8-dimethoxyflavone affected plasma membrane integrity of *B. cinerea* (Figure 5). In negative control, (dichloromethane), nuclei exhibited no fluorescence (Figure 5A). When hyphae were treated with ethanol (positive control) fluorescent nuclei were observed indicating alteration of the membrane (Figure 5B). After 4 h of incubation with 80 µg/mL of flavone alteration of the plasma membrane of *B. cinerea* was observed (Figure 5C); at lower concentration SYTOX staining was not produced (data no shown). After 6 h of incubation with the flavone, alteration of plasma membrane was produced at 40 or 80 µg/mL (Figure 5 D and E).

**Figure 6:**
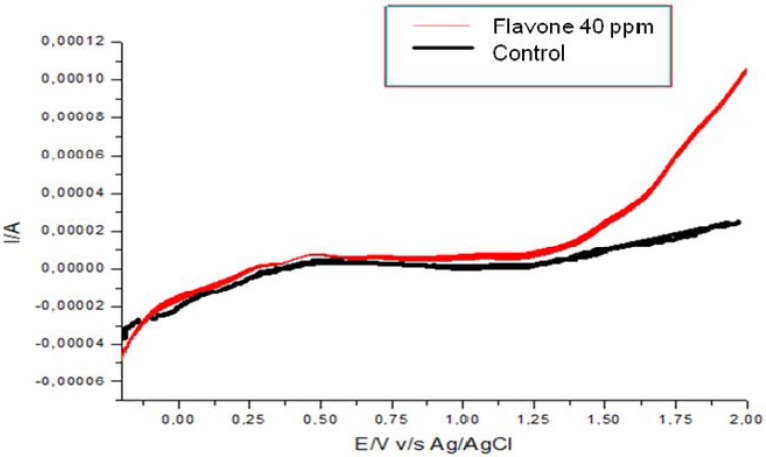
Voltammogram of 5,7-dihydroxy-3,8-dimethoxyflavoneat 40 μg/mL obtained at a scan rate of 0.1 V/s.

The effect on the oxygen consumption could be due to the compound's ability to interact with membranes and a direct interaction with the electron transporters would not occur. On the other hand, inhibitory activity on respiratory chain by some flavonoids has been associated with the oxidation potentials of these compounds [23]. This redox activity may generate reactive oxygen species in the mitochondria, therefore these flavonoids can be considered as potential pro-oxidants. The ability of purified flavonoid to act *in vitro* as antioxidant or pro-oxidant was thus evaluated (Figure 6). The flavone showed an oxidation wave over 1 V *vs.* Ag/AgCl. This voltammetric oxidation suggests that the flavone could indeed act as pro-oxidant [4]. In fact, according to Simic *et al.* [4] antioxidant behavior corresponds to species that present oxidation processes at potentials lower than ca. 0.45 V *vs.* Ag/AgCl. Species that showed oxidation processes at low potentials simultaneously they inhibited the lipid peroxidation [4]. The pro-oxidant activity of flavonoids can generate semiquinones and superoxide. Due to the very reactive nature of these radicals, macromolecules like proteins or DNA can be damaged [24,25]. In order to have good radical scavenging behavior, the flavonoid structure must allow a good electron delocalization and stabilization of the phenoxy radical [4,24]. According to the results observed in the voltammogram, where an ill-defined irreversible oxidation process took place, it is clear that the flavone was not able to stabilize the radical formed and therefore it was not able to act as an antioxidant. 

## 3. Experimental

### 3.1. Bioassay-guided Fractionation of Resinous Exudate from Pseudognaphalium Robustum

The resinous exudate was obtained by dipping the fresh plant material in cold dichloromethane for 20 s and the crude extract was concentrated in a rotary evaporator. A flavonoid with antifungal activity against *B. cinerea* was purified from the crude extract. Crude extract (5 g) was subjected to flash absorption chromatography and eluted with a hexane–ethyl acetate gradient. Twelve 200 mL fractions were collected and analyzed by thin layer chromatography (TLC). The fractions were pooled into five new fractions by chromatographic similarity. According to an antifungal assay against *B. cinerea*, one fraction (F3) was submitted to a new column chromatography applying 1.5 g of this fraction to an absorption column and eluting with a hexane–ethyl acetate gradient. A total of 120 fractions of 20 mL were collected and grouped into four fractions according to their chromatographic similarity. Fraction 76 was further purified by thin layer semipreparative chromatography in a CHCl_3_/acetone (98:2) system, to give a pure product (Fraction 76-2). Its structure was determined by ^1^H-NMR spectroscopy and co-elution with standards by high-performance liquid chromatography (HPLC) using a 250 × 4.6 mm Symmetry C18 5 µm column (Waters, MA, USA). The mobile phase was a linear gradient from 10% to 100% methanol-water with 1% (v/v) acetic acid over 45 min at a flow-rate of 1.0 mL/min. Eluents were monitored at 254 and 360 nm. NMR spectra were acquired using a Bruker Avance RW- 400 spectrometer operating at 400.13 MHz (1 h). Measurements were carried out at a probe temperature of 300 k, using CDCl3 containing tetramethylsilane (TMS) as an internal standard. 

### 3.2. Fungal Isolate and Culture Conditions

In this study *B. cinerea* strain T50 was used. This strain was originally isolated from naturally infected tomatoes (*Lycopersicon esculentum* cv. Roma) [27] and was maintained on malt-yeast extract agar slants with (2% (w/v) malt extract, 0.2% (w/v) yeast extract and 1.5% (w/v) agar) at 4 °C. The fungus was grown in the dark on malt-yeast extract agar medium or soft agar (2% (w/v) malt extract, 0.2% (w/v) yeast extract and 0.6 % (w/v) agar. Also, the following liquid minimum medium was used: (KH_2_PO_4_ (1 g/L), K_2_HPO_4_ (0.5 g/L), MgSO_4_∙7H_2_O (0.5 g/L), KCl (0.5 g/L), FeSO_4_∙7H_2_O (0.01 g/L), pH 6.5. Ammonium tartrate (25 mol/L) and glucose (1% (w/v)) were used as nitrogen and carbon source, respectively. All experiments were done at least in triplicate.

### 3.3. Effect of Column Fractions and Pure Compound on *B. cinerea*

The fungitoxicity of column fractions and pure compound against *B. cinerea* was assessed *in vitro* using the radial growth test on malt-yeast extract agar [28]. Fractions and pure compound were dissolved in dichloromethane at different final concentrations. Aliquots of these solutions (100 μL) were added to malt-yeast extract agar medium (5 mL). Final solvent concentration was identical in the control and treatment assays. Dichloromethane was allowed to evaporate prior to inoculation. The commercial fungicide iprodione dissolved in methanol, was used as control. Results of antifungal effect of purified compound and iprodione were expressed as effective dose (ED_50_; the concentration that reduced mycelial growth by 50%) determined by regressing the inhibition of radial growth values (percent control) after 96 hours of incubation against compound concentration. All experiments were done at least in triplicate. 

Also, the effect of pure compound on conidial germination was determined. Conidial germination assays were carried out on microscope slides coated with soft agar medium (2 mm thickness). Pure compound was added dissolved in dichloromethane at a final concentration of 40 μg/mL. Dichloro-methane was allowed to evaporate prior to inoculation. The slides were inoculated with dry conidia obtained from sporulated mycelia (1 week old), placed in a humid chamber (90% relative humidity), and incubated in the dark at 22 °C for 7 h. Conidial germination was determined directly on the slides at 1-hour intervals. The percentage of germination was estimated by counting the number of germinated conidia in five microscope fields each containing approximately 40 conidia. Conidia were judged to have germinated when the germ tube length was equal to or greater than conidial diameter. Each experiment was done at least in triplicate.

### 3.4. Effect of Pure Compound on the Membrane Integrity of *B. cinerea*

This was determined using the SYTOX Green uptake assay [29]. *B. cinerea* conidia at a final concentration of 1 × 10^5^ conidia/mL were inoculated in 24-well plates (lined with 12-mm glass coverslips) containing 1 mL of liquid minimum medium. Cultures were incubated at 22 °C for 15 h to permit the germination of the conidia. After this time, liquid medium was removed and same medium with 70% (v/v) ethanol (positive control), 2% (v/v), dichloromethane (negative control), or 40 and 80 μg/mL of purified flavone was added to each well. The mixtures were incubated at 22 °C for one, four and six hours in the case of flavone and dichloromethane or for 10 min when ethanol was used. *B. cinerea* hyphae adhered to glass coverslips were washed three times with liquid minimum medium and were stained with 50 nM SYTOX Green (Molecular Probes, Eugene, OR, USA). After 10 min of incubation, the hyphae were washed with minimum medium and glass coverslips containing hyphae were mounted in slides. For the assembly of the samples in the slides, 15 µL of DABCO (1,4-diazabicyclo[2.2.2]octane) was used. The fluorescence of *B. cinerea* hyphae stained with SYTOX Green was observed under a confocal microscope (Carl Zeiss LSM 510) at an excitation wavelength of 488 nm and an emission wavelength of 540 nm. These experiments were done at least in triplicate.

### 3.5. Effect of Pure Compound on the Oxygen Consumption of *B. cinerea* Conidia

Oxygen consumption was determined polarographically at 25 °C with a Hansatech oxygen electrode, using germinating conidia in a total volume of 1 mL [14]. To obtain conidia in suspension, Murashige and Skoog's basal medium at 4.4 g/L (Phytotechnology Laboratories, Lenexa, KS, USA) was added to Petri dishes containing conidia. The conidia were harvested by scraping with a sterile spatula. To eliminate mycelia, the suspension was filtered through glass wool. Conidia concentration was adjusted to 1 × 10^7^ conidia/mL with liquid minimum medium, in the presence of 2% (w/v) glucose. Conidia were incubated for 2 hours at 22 °C. Measurement of basal oxygen consumption was made for 2 min in the same liquid minimum medium. After that time, carbonyl cyanide *m*-chlorophenylhydrazone (CCCP, 10 μg/mL), KCN (650 μg/mL), or pure compound dissolved in dichloromethane at 40 or 80 μg/mL were added. Oxygen consumption was determined for eight more minutes. As negative control, dichloromethane (at identical concentration of treatment assays) was used. KCN and CCCP were used as inhibitor or uncoupler of respiratory chain, respectively. 

### 3.6. Pro-oxidant Activity

Pro-oxidant effect of pure compound was determined using cyclic voltammetry as has been described [4]. Electrochemical experiments were performed in a three-compartment glass cell, glassy carbon (A = 0.071 cm^2^) was used as working electrode. A saturated Ag/AgCl/KCl(sat) was used as reference electrode with respect to which all the potentials are quoted, and a Pt coil (A = 14 cm^2^) was used as the counter electrode. The glassy carbon electrode was polished with 0.25 µm alumina and ultrasonicated for 5 min before each experiment. Pro-oxidant behavior was determined by potentiodynamically cycling the electrode between −0.2 V and 2 V at a scan rate of 0.1 V/s in dichloromethane containing 40 or 80 μg/mL of the flavone in the presence of 0.1 M tetrabutylammonium perchlorate. The solution was purged with nitrogen (ultra pure grade) during each measurement. All the experiments were carried out at room temperature in an inert atmosphere. Electrochemical measurements were performed using an AFCBP1 Pine Bipotentiostat, along with Pinechem 2.5 software.

## 4. Conclusions

The flavonoid purified in this study, 5,7-dihydroxy-3,8-dimethoxyflavone, is active *in vitro* against *B. cinerea*, affecting mycelia growth and conidia germination. This compound interacts with the plasma membrane of *B. cinerea* and it affects the mitochondrial respiratory chain. Effects against *B. cinerea* would be due to its pro-oxidant properties.
